# The Trembles and Milk Sickness

**Published:** 1842-03

**Authors:** 


### THE WESTERN JOURNAL.

Vol. V.—No. III.

LOUISVILLE, MARCH 1, 1842.

---

### THE TREMBLES AND MILK SICKNESS.

Some ten or twelve months since we published in the Journal a memoir on these maladies, as they have prevailed *between the Little Miami and Sciota rivers, in the State of Ohio.* In this memoir we included nothing previously published by any person concerning those diseases in that quarter, much less any where else, nor did we introduce any of our own unpublished remarks made in other parts of the West. It was in fact a monograph of those diseases, as they occur in *that* particular district; and all the conclusions at which we arrived, were from the facts collected while exploring it. In no case whatever, did we inquire whether these conclusions were either confirmed or invalidated, by facts observed beyond the narrow limits prescribed in our paper. Of the conclusions to which we refer, the most important were, that certain localities within the district generated the Trembles, while others are exempt; that in the former there are numerous scions from the roots of the climbing *Rhus radicans* of Linnæus, and are known by the people under the name of poison oak; that the destruction of the natural vegetation of these spots puts a stop to the further progress of the disease; and that the facts collected show that this species of *rhus may* be, but do not prove that it *is*, the cause of Trembles.

Now, it is really amusing to see what a stir these and some other incontrovertible *facts*, in the same memoir, have raised among the milk-sick-literati of the West, each one of whom is nursing a special hypothesis, for which he is not only willing but desirous of fighting.

Some of them have merely heard of our memoir—others have seen, and misunderstood its plainest statements—others have wilfully misquoted and misstated its facts! Not one has visited the district, and ascertained that the memoir embraces a single false observation! Some have discovered that we are visionary and speculative, although we consulted more than a hundred persons, before admitting the fact, that calves experience the Trembles from the milk of their dams, and that dogs die from eating the flesh of animals dead from Trembles. We would like to know which of our sick-stomach-literateurs, has devoted a fortnight of diligent personal inquiry to the ascertainment of those facts? Our inquiries about the *rhus radicans* were conducted with equal patience and caution; and at the close we asked whether all the facts collected "*prove*" it to be the cause of Trembles; to which the answer given in the next line is, "*certainly not.*" Notwithstanding all this, we are charged with having declared this plant the cause! and then abused for loose observation, wild theorising, ignorance and falsehood! The worthies who agree in all this, disagree, however, in every thing else, for while each is showing off his own nursling, he is running down that of his rival. We congratulate the gentlemen on having such a fountain of excitement, now when the temperance reform has nearly closed up the alcoholic; and are not quite so misanthropic as to prevent them from misrepresenting and abusing us, whenever they can find enjoyment in it. All we ask, is the privilege of telling the public, now and then, that not being able to controvert what we have written, they have undertaken to controvert that which we have not written. D.

